# Glucocorticoid-Induced Leucine Zipper (GILZ) Antagonizes TNF-α Inhibition of Mesenchymal Stem Cell Osteogenic Differentiation

**DOI:** 10.1371/journal.pone.0031717

**Published:** 2012-03-02

**Authors:** Linlin He, Nianlan Yang, Carlos M. Isales, Xing-Ming Shi

**Affiliations:** 1 Institute of Molecular Medicine and Genetics, Georgia Health Sciences University, Augusta, Georgia, United States of America; 2 Faculty of Life Sciences, Northwestern Polytechnical University, Xi'an, China; 3 Department of Orthopaedic Surgery, Georgia Health Sciences University, Augusta, Georgia, United States of America; 4 Department of Pathology, Georgia Health Sciences University, Augusta, Georgia, United States of America; University of Southern California, United States of America

## Abstract

Tumor necrosis factor-alpha (TNF-α) is a potent proinflammatory cytokine that inhibits osteoblast differentiation while stimulating osteoclast differentiation and bone resorption. TNF-α activates MAP kinase pathway leading to inhibition of osterix (Osx) expression. TNF-α also induces the expression of E3 ubiquitin ligase protein Smurf1 and Smurf2 and promotes degradation of Runx2, another key transcription factor regulating osteoblast differentiation and bone formation. We showed previously that overexpression of glucocorticoid (GC)-induced leucine zipper (GILZ) enhances osteogenic differentiation of bone marrow mesenchymal stem cells (MSCs). We and others also showed that GILZ is a GC effecter and mediates GC anti-inflammatory activity. In this study, we asked the question whether GILZ retains its osteogenic activity while functioning as an anti-inflammatory mediator. To address this question, we infected mouse bone marrow MSCs with retroviruses expressing GILZ and induced them for osteogenic differentiation in the presence or absence of TNF-α. Our results show that overexpression of GILZ antagonized the inhibitory effects of TNF-α on MSC osteogenic differentiation and the mRNA and protein expression of Osx and Runx2, two pivotal osteogenic regulators. Further studies show that these antagonistic actions occur via mechanisms involving GILZ inhibition of TNF-α-induced ERK MAP kinase activation and protein degradation. These results suggest that GILZ may have therapeutic potential as a novel anti-inflammation therapy.

## Introduction

Chronic inflammation, such as rheumatoid arthritis (RA), causes bone loss. Long-term use of steroid hormone glucocorticoids (GCs), which are potent anti-inflammatory agents and are frequently used to treat these conditions, also causes bone loss and results in osteoporosis (GC-induced osteoporosis), and is thus a major limiting factor for long term GC therapy [1], [2]. Despite intensive investigations, the molecular mechanisms underlying GC anti-inflammatory actions and GC-induced bone loss are still not clear.

Bone marrow mesenchymal stem cells (MSCs) are multipotent cells and can differentiate into several distinct cell lineages, including bone-forming osteoblasts and cartilage-forming chondrocytes [3]. Several recent studies have reported that MSCs are present in cultures of RA synovial fluid or tissue [4]–[7]. These MSCs are believed to be recruited to the arthritic joints but, due to the inflammation, their normal differentiation is arrested and play an important role in the pathogenesis of RA [8].

The osteogenic differentiation of MSC is controlled by two key transcription factors, osterix (Osx) and Runx2 [9], [10]. The expression of Osx and Runx2 are regulated by many factors such as hormones/growth factors and cytokines including tumor necrosis factor-alpha (TNF-α), one of the major proinflammatory cytokines whose production is elevated in arthritic joints and causes inflammation, cartilage destruction, and bone erosion [11], [12]. Transgenic mice overexpressing TNF-α are severely osteoporotic [13], and blockade of TNF-α by its soluble receptor (Etanercept) effectively reduces the inflammatory response. Studies have shown that key mechanisms by which TNF-α inhibits Osx and Runx2 include activation of the mitogen-activated protein kinase (MAPK) pathway and induction of the E3 ubiquitin ligase Smurf proteins. Activation of ERK MAP kinase by TNF-α results in inhibition of Osx transcription [14], and induction of Smurf1 and Smurf2 results in accelerated Runx2 protein degradation through the proteasomal degradation pathway [15]–[17].

Glucocorticoid-induced leucine zipper (GILZ) is a member of the leucine zipper protein family and belongs to the transforming growth factor β-stimulated clone-22 (TSC-22d3) family of transcription factors [18]. GILZ can physically interact with and inhibit the activities of the key inflammatory signaling mediators NF-κB and AP-1 [19], [20]. GILZ also interacts with MAP kinase family members Ras and Raf, resulting in inhibition of Raf-1 phosphorylation and, subsequently, inhibition of ERK1/2 phosphorylation and AP-1-dependent transcription [21], [22].

We reported previously that overexpression of GILZ in bone marrow MSCs enhances osteogenic differentiation by shifting MSC lineage commitment towards the osteoblast pathway [23]. We also showed that GILZ is a GC effect mediator and inhibits TNF-α-induced cyclooxygenase-2 (Cox-2) expression by blocking TNF-α-induced NF-κB nuclear translocation [24]. In this report we show that GILZ antagonizes the inhibitory effect of TNF-α on MSC osteogenic differentiation and provide a possible mechanism by which it does so. Since GILZ is an anti-inflammatory molecule and pro-osteogenic, this study suggests that GILZ could be an ideal drug candidate for a new anti-inflammatory therapy.

## Results

### Overexpression of GILZ antagonizes the inhibitory effect of TNF-α on MSC osteogenic differentiation

**Figure 1:**
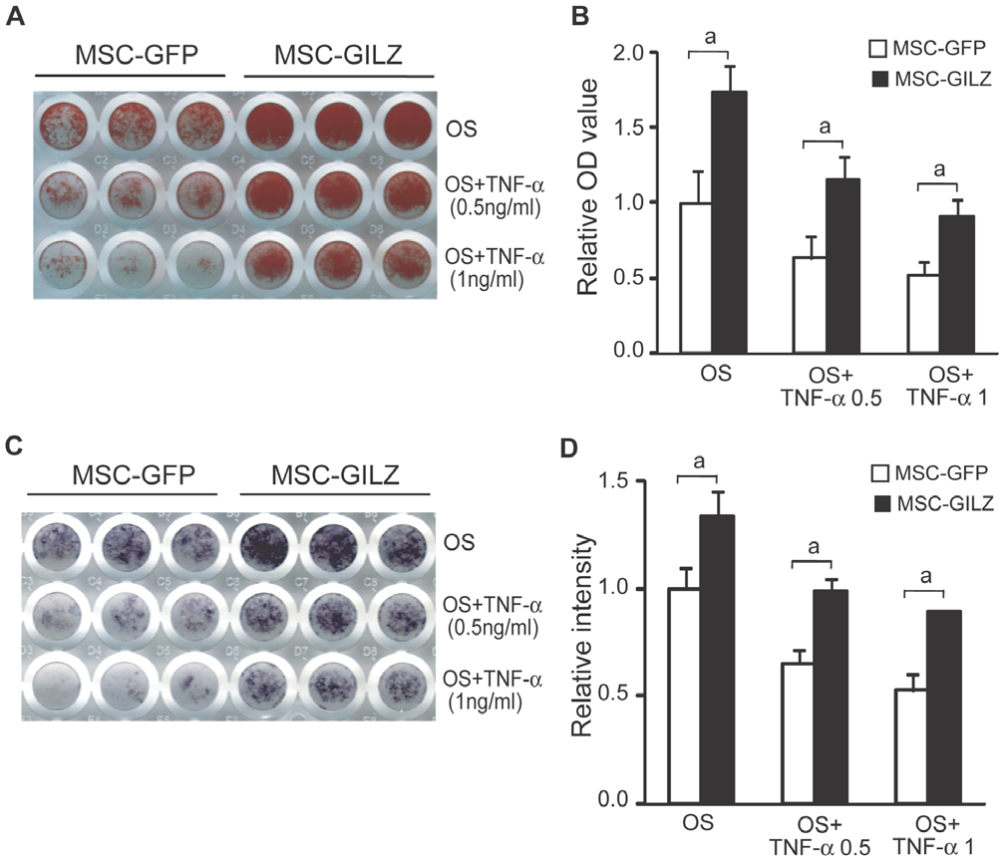
Effect of GILZ on TNF-α inhibition of MSC osteogenic differentiation. MSC-GFP and MSC-GILZ cells were cultured in osteogenic induction media (OS) in the absence or presence of indicated concentrations of TNF-α. (A) Cells were stained with alizarin red S on day 21 to visualize mineralized bone matrix. (B) Bar graph showing quantitative results of A. After scanning, the plates were destained with a solution containing 10% cetylpyridinium chloride in sodium phosphate. Concentrations of recovered ARS dye were determined by absorbance measurement and presented as fold changes relative to the OD value of MSC-GFP cells cultured in OS media. Each bar represents an average of 15 wells from 3 independent plates. (C) MSC-GFP and MSC-GILZ cells were cultured as above (A) for 8 days and stained for ALP to detect surface ALP-positive cell populations. (D) Bar graph showing quantitative results of C. Intensities of scanned images were analyzed with NIH Image J software and presented as fold changes relative to the value of MSC-GFP cells cultured in OS media. Each bar represents an average of 15 wells from 3 independent plates. Representative scanned images are shown. Error bars indicate S.D. a, *p*<0.01.

To investigate whether GILZ is capable of enhancing MSC osteogenic differentiation while serving as an anti-inflammatory mediator, we infected MSCs with retroviruses expressing GILZ or GFP (MSC-GILZ and MSC-GFP, respectively) and induced them with osteogenic induction media (OS) in the absence or presence of different concentrations of TNF-α. Results show that in the absence of TNF-α, both MSC-GILZ and MSC-GFP cells differentiated normally and formed mineralized bone nodules after 21 days of culture as indicated by alizarin red S (ARS) staining (Fig. 1A, first row). In the presence of TNF-α, however, mineralization of MSC-GFP control cells was inhibited in a TNF-α dose-dependent manner (Fig. 1A, second and third rows). In contrast, this inhibition was largely prevented in MSC-GILZ cells. Same result was obtained by alkaline phosphatase (ALP) staining of a shorter term (8 days) culture under the same conditions (Fig. 1C). Higher concentrations of TNF-α (>3 ng/ml) cause the death of both MSC-GILZ and MSC-GFP cells in long-term culture. Consistent to our previous studies, MSC-GILZ cells always showed a significantly higher degree of mineralization than the MSC-GFP cells (Fig. 1A, first row) [23]. Quantitative results of Fig. 1A and C are shown as bar graphs in Fig. 1B and D, respectively. Because dexamethasone, which induces endogenous GILZ, was not supplemented in our osteogenic induction media, these results demonstrate that the anti-TNF-α effect observed here was due to the overexpression of GILZ.

### GILZ antagonizes TNF-α inhibition of osterix (Osx) and Runx2

**Figure 2:**
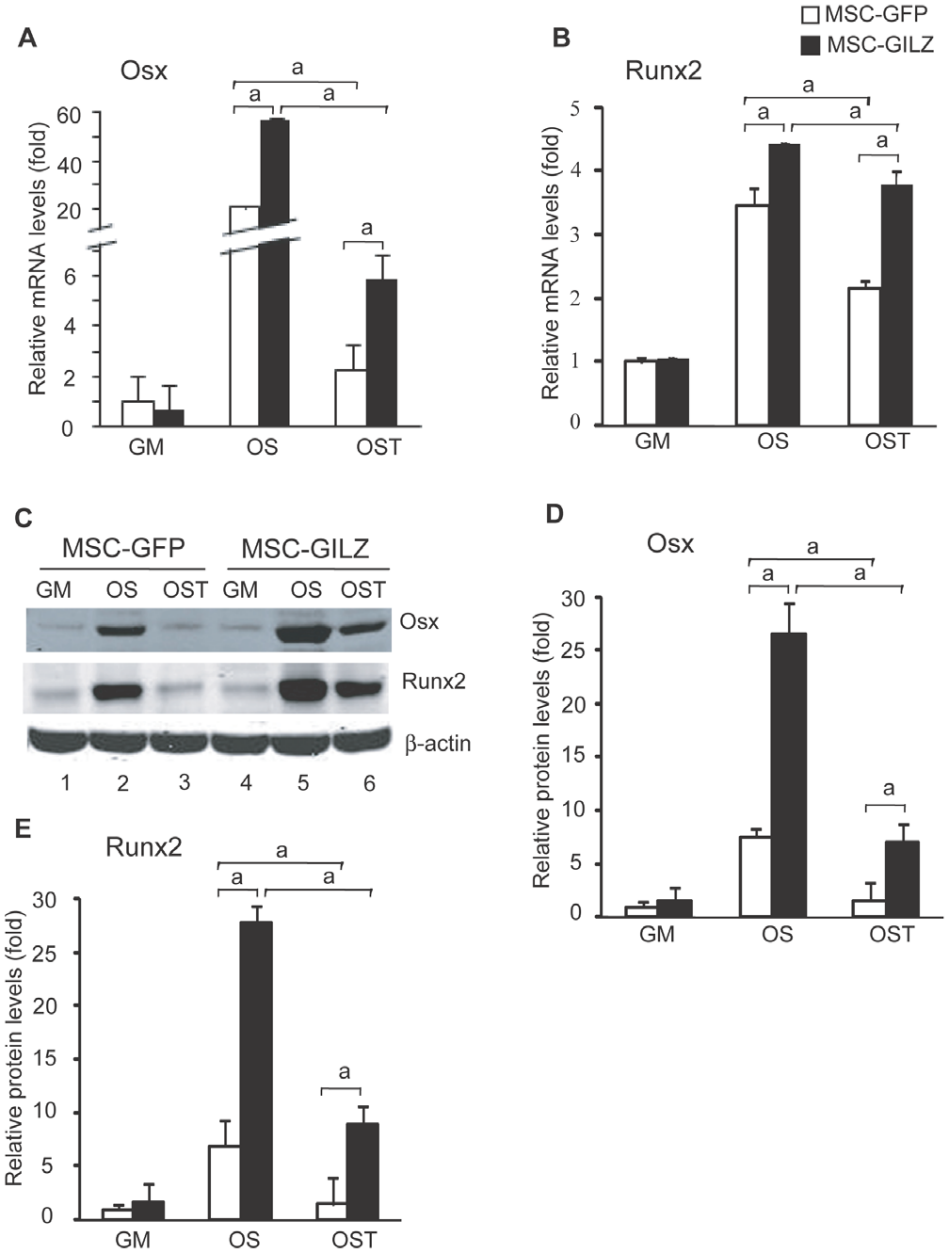
Effect of GILZ on TNF-α regulation of Osx and Runx2 expression. Real-time qRT-PCR and Western blot analyses of mRNA and protein expression. MSC-GFP and MSC-GILZ cells were cultured in OS with or without 1 ng/ml TNF-α (OST) for 8 days. At the end of treatment, one set of cells was harvested for real-time qRT-PCR (A, B) and a second set for Western blot (C) analyses of Osx and Runx2. Equal loading of samples in each lane is shown by the levels of β-actin. These experiments were performed at least three times with similar results. Quantitative results for levels of Osx and Runx2 protein are presented as bar graphs (D, E). Value of intensity for MSC-GFP cells cultured in regular DMEM growth medium (GM) is arbitrarily set as 1. Error bars indicate S.D. a, *p*<0.01 (n = 3).

To elucidate the molecular mechanisms underlying GILZ antagonizing TNF-α inhibition of osteoblast differentiation, we examined the expression levels of Osx and Runx2 mRNA and protein. MSC-GFP and MSC-GILZ cells were cultured in OS in the absence or presence of TNF-α (1 ng/ml). Eight days after the treatment, one set of cells was harvested for RNA analysis and the other set for protein analysis. Results show that in the absence of TNF-α, levels of Osx and Runx2 mRNA (Fig. 2A, B) and protein (Fig. 2C–E) were induced significantly in both GFP- and GILZ-expressing cells. TNF-α treatment reduced the levels of Osx and Runx2 in both MSC-GFP and MSC-GILZ cells. However, compared with MSC-GFP cells, the levels of Osx and Runx2 mRNA and protein were still significantly higher in MSC-GILZ cells.

### GILZ inhibits TNF-α-induced ERK phosphorylation and Smurf expression

**Figure 3:**
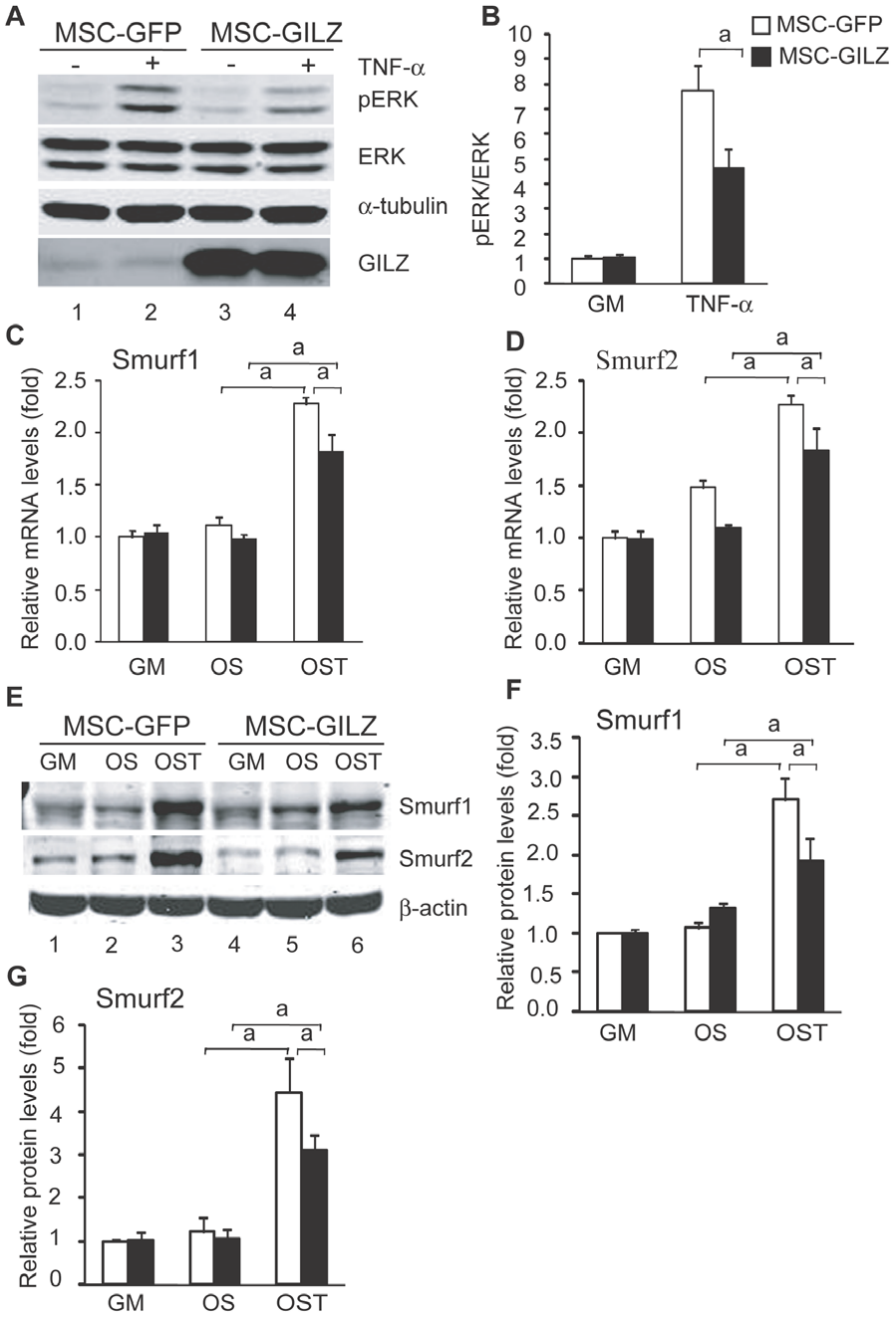
Effect of GILZ on TNF-α-induced ERK phosphorylation and Smurf expression. (A, B) MSC-GFP and MSC-GILZ cells were serum starved for 48 hr and then treated with TNF-α (1 ng/ml) for 15 min before harvesting. Equal amounts of total cell lysates were separated on SDS PAGE gel, transferred onto membrane and analyzed for levels of phosphorylated ERK1/2 (pERK). Levels of pERK were quantified with NIH Image J software and presented as a ratio of pERK/total ERK in B. Value from untreated MSC-GFP is arbitrarily set as 1. Experiments were repeated three times with similar results. Levels of overexpressed GILZ are also shown. Error bars indicate SD. α-tubulin, instead of β-actin, was used as a loading control in these experiments because β-actin and ERK have similar molecular weights. (C–F) RNA and protein samples from MSC-GFP and MSC-GILZ cells used in the experiments described in figure 2 were analyzed by real-time qRT-PCR and Western blot to show the levels of Smurf1 and Smurf 2 mRNA (C, D) and protein (E). Quantitative results of Fig. 3E are shown as bar graphs in Fig. 3F and G, respectively. Data were normalized to β-actin and expressed as fold changes relative to control cells cultured in GM. These experiments were repeated three times with similar results. Error bars indicate SD. a, *p*<0.01 (n = 3).

To determine whether the protection of Osx by GILZ (Fig. 2) was due to the inhibition of TNF-α-induced ERK/MAP kinase activity, we treated cells with TNF-α (1 ng/ml, 15 min) and examined the status of ERK phosphorylation using an antibody specific to phosphorylated ERK. Results show that TNF-α treatment induced ERK1/2 phosphorylation in both MSC-GFP and MSC-GILZ cells; however, this induction was reduced significantly in MSC-GILZ cells (Fig. 3A, compare lane 4 to lane 2). GILZ seems to have no significant effect on basal levels of ERK1/2 phosphorylation (compare lane 1 to lane 3, and the quantitative results in Fig. 3B). Levels of total ERK were detected by antibodies against ERK. Equal loading of lanes is shown by levels of α-tubulin. Together, these results demonstrated that overexpression of GILZ can inhibit TNF-α-induced ERK/MAP kinase activation, and suggest that GILZ antagonizes TNF-α inhibition of Osx, in part, by inhibiting ERK1/2 phosphorylation.

Next, we examined the levels of E3 ubiquitin ligases Smurf1 and Smurf2 since studies have shown that TNF-α up-regulates Smurf1 and Smurf2, which, in turn, promotes Runx2 protein degradation [15], [16]. The same RNA samples collected from cells treated with TNF-α for analysis of Osx and Runx2 mRNA described above (Fig. 2) were used. Results show that overexpression of GILZ (MSC-GILZ) reduced TNF-α-induced Smurf1 and Smurf2 expression at both mRNA (Fig. 3C, D) and protein (Fig. 3E–G) levels.

### Effect of GILZ on Osx promoter activity

**Figure 4:**
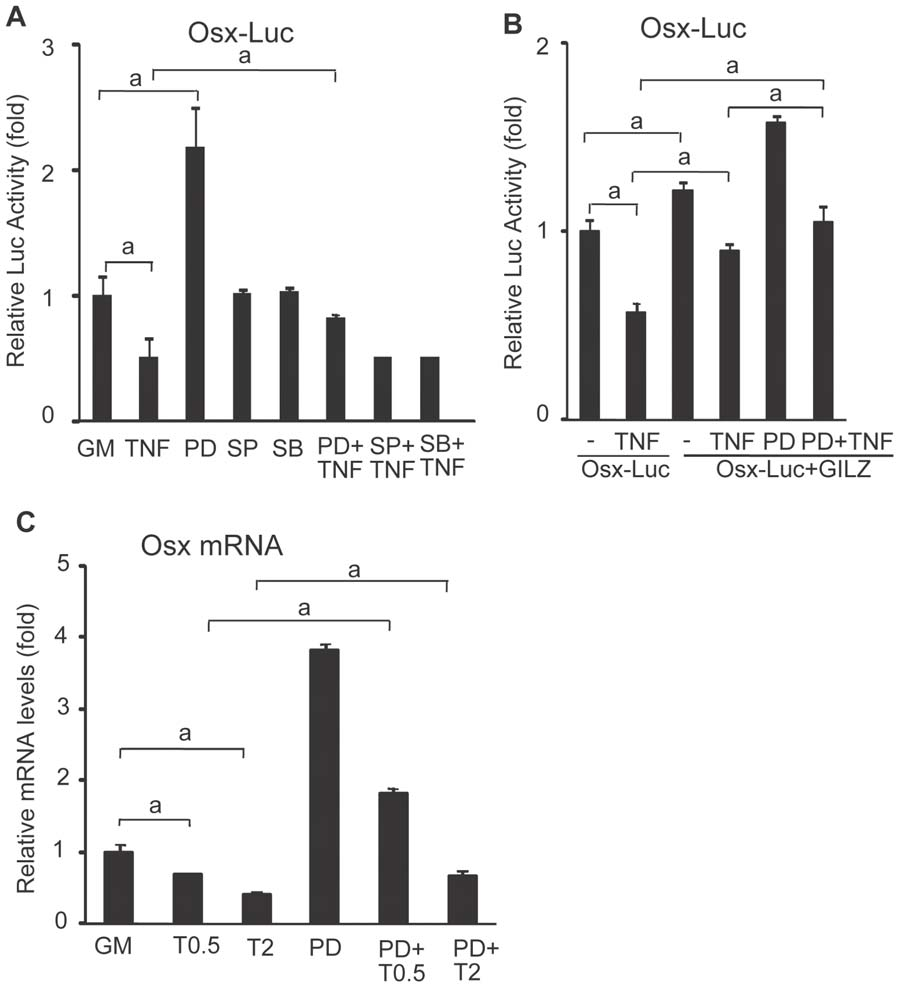
Effect of GILZ on TNF-α regulation of Osx transcription. (A, B) Transient transfection and luciferase-reporter assays. (A) C3H10T1/2 cells were transfected with Osx-Luc promoter reporter and treated either with TNF-α (10 ng/ml) or an indicated kinase inhibitor alone, or with TNF-α plus an inhibitor for 24 hr and then harvested for luciferase assays. The concentration of TNF-α used in this experiment is based on the study by Lu et al [14] as the promoter reporter construct is same. (B) C3H10T1/2 cells were cotransfected with Osx-Luc reporter and a GILZ expression plasmid and treated with TNF-α and ERK inhibitor PD98059 as indicated. Luciferase activity was measured as in A. these experiments were performed 3 times in triplicate. (C) Real-time qRT-PCR analysis. MSCs were first cultured in OS medium for 5 days (to initiate the osteogenic differentiation program) and then were treated continuously with PD98059 for 17 hr, or treated with PD98059 for 5 hr prior to TNF-α (0.5 or 2 ng/ml, T.5 and T2, respectively) treatment for 12 hr before harvesting for RNA isolation and qRT-PCR analysis. Data were normalized to β-actin and expressed as fold changes relative to the mRNA level of untreated (GM) Cells. Experiments were performed 2 times in duplicates with the same results. Error bars indicate SD. a, *p*<0.01 (n = 2).

Since GILZ antagonizes TNF-α-induced ERK phosphorylation (Fig. 3), an event through which TNF-α regulates Osx expression, we next examined whether this antagonism occurs at the transcriptional level. A 791-nucleotide fragment of the Osx promoter region (−700 to +91 relative to the transcription start site) was PCR amplified from mouse genomic DNA and inserted into a luciferase reporter vector (pGL3-basic, see details in Methods). This reporter construct, referred to as Osx-Luc, was transfected into C3H10T1/2 cells. The transfected cells were treated with TNF-α or different kinase inhibitors separately, or TNF-α plus an inhibitor, to determine which kinase pathway is primarily responsible for TNF-α-mediated inhibition of Osx. As with the mRNA and protein expression profiles, TNF-α reduced basal level of Osx-Luc reporter activity significantly (Fig. 4A, compare column 2 with column 1). Treatment of cell with PD98059 (ERK inhibitor) significantly increased Osx-Luc reporter activity (compare column 3 with column 1) and neutralized TNF-α inhibition on this reporter activity (compare column 6 with columns 1 and 2). In contrast, SP600125 (JNK inhibitor) or SB203580 (p38 inhibitor) had no such an effect (compare columns 4 and 5 with column 1; columns 7 and 8 with column 2). These results demonstrated that TNF-α inhibits Osx promoter transcriptional activity through activating ERK/MAP kinase pathway, but not JNK or p38 pathway. To determine the role of GILZ in ERK phoshorylation, and subsequently, Osx transcription, we co-transfected C3H10T1/2 cells with Osx-Luc reporter and a GILZ expression plasmid. Results show that overexpression of GILZ, which, by itself enhanced Osx-Luc basal promoter activity (Fig. 4B, compare column 3 with column 1), counteracted TNF-α inhibitory effect and rescued the promoter activity almost to its base level (compare column 4 with columns 1 and 2). Treatment of cells with PD98059 offset completely the TNF-α effect and restored Osx-Luc promoter activity to its basal level (compare column 6 with columns 1 and 2), but overexpression of GILZ did not synergize or enhance PD98059 effect (compare column 5 with column 3). These results demonstrated that the antagonistic effect of GILZ on TNF-α inhibition of Osx is fulfilled, in part, through GILZ inhibition of TNF-α-induced ERK1/2 phosphorylation. This conclusion is further supported by the changes of Osx mRNA expression in MSCs treated with TNF-α and ERK inhibitor, which showed that PD98059 rescues TNF-α-mediated inhibition of Osx mRNA expression (Fig. 4C).

## Discussion

In this study we have demonstrated that GILZ antagonizes the inhibitory effect of TNF-α on MSC osteogenic differentiation. Evidence presented in this study showed that GILZ antagonizes TNF-α effect in osteoblast differentiation by inhibiting TNF-α-induced ERK/MAP kinase activation and by inhibiting the expression of E3 ubiquitin ligase Smurf proteins. This antagonistic effect resulted in a partial rescue of TNF-α inhibition on Osx transcription, and a deceleration of TNF-α-induced Runx2 degradation. Attempts to confirm these results by knocking down GILZ were proven to be unsuccessful due to the low basal levels of endogenous GILZ expression (barely detectable by Western blot), and approaches that can increase GILZ levels, such as treatment of cells with glucocorticoid, activates glucocorticoid receptor and induces the expression of MAP kinase phosphatase-1 (MKP-1) [25], which inhibits ERK phosphorylation and compensates GILZ knockdown effect. We think that the pro-osteogenic effect of GILZ is overridden by the glucocorticoid receptor in the continued glucocorticoid presence and that the adverse bone effect of glucocorticoids is mediated primarily by glucocorticoid receptor. Supporting this notion, a recent *in vivo* study showed that mice with bone-specific glucocorticoid receptor knockout resist to glucocorticoid-induced bone loss [26]. Indeed, continuous dexamethasone treatment, which activates glucocorticoid receptor constantly, reduces mineralized bone nodule formation (Figure S1). We think that our current system is inadequate for testing GILZ knockdown effect. A better experimental system, such as knockdown GILZ in glucocorticoid receptor knockout or MKP-1 knockout MSCs, should be appropriate for addressing this issue.

Osx and Runx2 are pivotal transcription factors regulating osteogenic differentiation and bone formation. These two genes are primary targets for TNF-α in osteoblasts. In addition to activating the ERK pathway, TNF-α also activates NF-κB and induces p65/NF-κB nuclear translocation [19], [24]. However, elegant experiments carried out in the Nanes laboratory have demonstrated that the inhibition of Osx by TNF-α does not involve the NF-κB pathway, although TNF-α induced p65 nuclear entry and TNF-response element is present in the Osx promoter region [14]. In fact, forced overexpression of NF-κB activated Osx promoter activity (*in vitro* luciferase reporter assays) [14]. Since NF-κB pathway does not play a major role in TNF-α-mediated inhibition of Osx, and our previous studies have shown that GILZ inhibits TNF-α-induced p65/NF-κB nuclear translocation in MSCs [24], we did not pursue this part of the study further because these studies were conducted in the same experimental system.

The inhibition of Runx2 by TNF-α, on the other hand, is primarily post-translational, involving TNF-α up-regulation of Smurf proteins and resulting in accelerated Runx2 protein degradation [16], [17]. A perfect AP-1 motif is found in the Runx2 promoter region. Although other AP-1 members, i.e., Fra2 and JunD, can bind to this site, c-Jun and c-Fos, with which GILZ interacts, showed no binding activity [27]. These published studies demonstrated that the accelerated Runx2 protein degradation, which is triggered by TNF-α induction of Smurf expression, is a key mechanism by which TNF-α regulates Runx2 activity. NF-κB seems to involve only in the inhibition of BMP signaling by TNF-α. Activation of NF-κB results in disruption of BMP-induced Smad DNA-binding activity [28].

Although the causes of rheumatoid arthritis (RA), which is characterized by inflammation, pain, and swelling [11], are still not clear, the levels of proinflammatory cytokines (i.e., TNF-α, IL-1β) and inducible enzymes involved in inflammation (i.e., COX-2) are significantly increased at the inflammation sites. Thus, inhibition of these inflammatory molecules, for example, by using TNF-α blocker (Etanercept) and COX-2 selective inhibitors (Coxibs), has been a common clinical practice. Glucocorticoids are among the best known anti-inflammatory agents and have been widely used in the clinic for treating RA [29]–[32]. However, long-term glucocorticoid therapy causes many side effects, including osteoporosis. This significant clinical problem was recognized almost the same time that glucocorticoids were introduced into the clinic nearly 60 years ago, and it has been a major factor for limiting long-term glucocorticoid therapy. Recent studies in our laboratory have shown that overexpression of GILZ enhances MSC osteogenic differentiation [23] and inhibits TNF-α-induced COX-2 expression [24]. Studies from other laboratories have also demonstrated that GILZ is a glucocorticoid anti-inflammatory action mediator [33]–[35]. Importantly, a recent *in vivo* study by Beaulieu et al showed that the levels of GILZ is increased by therapeutic doses of glucocorticoids in the synovium of collagen-induced arthritis (CIA) mice, and that knockdown of GILZ using siRNA increases the clinical and histologic severity of CIA and synovial expression of TNF-α and IL-1 [36]. This *in vivo* study directly supports our findings reported here regarding the role and therapeutic potential of GILZ in RA. TNF-α is a potent proinflammatory cytokine and triggers inflammatory reactions and further amplifies inflammatory signals by inducing other inflammatory cytokines (e.g., IL-1β, IL-6), chemokines (e.g., MIP-1α, MCP1), and inducible enzymes (e.g., COX-2, iNOS). Importantly, TNF-α inhibits genes that directly regulate osteoblast differentiation and bone formation [14]–[16], [37]. Thus, approaches that can inhibit TNF-α activity (production or action) would have therapeutic potential for preventing bone loss. Evidence that GILZ not only mediates glucocorticoid anti-inflammatory action, but also enhances MSC osteogenic differentiation suggests that GILZ is a prominent anti-inflammatory drug candidate that could be safe to bone.

## Materials and Methods

### Reagents and chemicals

TNF-α (#410-MT) was purchased from R&D Systems Inc.; PD98059 (#PHZ1164) from BioSource International, Inc.; One-Step NBT/BCIP solution (#34042) from Pierce; SYBR Green PCR Master Mix (#4367659) from Applied Biosystems; Osx antibody (#ab22552) from Abcam Inc.; Runx2 (#130-3) monoclonal antibody from MBL International; Smurf1 (#AP2104a) and Smurf2 (#AP2105a) antibodies from Abgent Inc.; ERK1/2 (#9102) and phospho-ERK1/2 (#9106) antibodies from Cell Signaling Technology, Inc. All chemicals, except where specified, were purchased from Sigma-Aldrich.

### Mouse bone marrow MSCs

Isolation, characterization and infection of MSCs were described previously [23], [24], [38]. Briefly, MSCs were isolated from 18-month-old male C57BL/6 mice using a negative immuno-depletion (using magnetic beads conjugated with anti-mouse CD11b, CD45R/B220, and Pan DC) and positive immuno-selection (using anti-Sca-1 beads) approach with an animal procedure approved by the Institutional Animal Care and Use Committee (IACUC) at the Georgia Health Sciences University (Approval #: BR08-10-108). For GILZ overexpression, the MSCs were infected with retroviruses expressing GILZ (MSC-GILZ) or GFP (MSC-GFP, as a control). The viral infection efficiency (>90%) and levels of GILZ expression in the infected cells were confirmed by Western blot and immunofluorescence microscopy as previously described [23], [24].

### Osteogenic differentiation

Cells were plated in 96-well plates in triplicate at a density of 1×10^4^ cells/cm^2^ the previous day and then treated with osteogenic supplements (OS) consisting of DMEM supplemented with 2% FBS, 5 mM β-glycerophosphate, and 50 µM L-ascorbic acid-2-phosphate for the indicated time period.

For alkaline phosphatase (ALP) staining, cells were rinsed 3 times with calcium and phosphate-free saline solution, fixed in 4% paraformaldehyde for 30 min at RT, washed 3 times with double-distilled water, and stained with One-Step™ NBT/BCIP solution (Pierce, Rockford, IL) for 15 min at RT. Cells were washed again with double-distilled water and the plates were scanned using a CanoScan LiDE 80 flat-bed scanner.

For alizarin red S (ARS) staining, cells were rinsed with calcium and phosphate-free saline solution, and fixed with ice-cold 70% ethanol for 1 hr. After a brief wash with water, cells were stained for 10 min with 40 mM ARS solution (pH 4.2) at room temperature. Cells were rinsed five times with water followed by a 15-min wash with PBS (with rotation) to reduce nonspecific ARS staining. After scanning of the plate for digital images, ARS dye was dissolved in 10% cetylpyridinium chloride (CPC) in 10 mM sodium phosphate (pH 7.0) for 15 min at RT and the concentrations of ARS determined by absorbance measurement as previously described [23].

### Western blot analysis

Cells were cultured in osteogenic induction media in the presence of TNF-α (1 ng/ml) for 8 days before they were harvested. Equal amounts of total protein were separated on SDS-PAGE and analyzed by Western blot as previously described [23], [24] using the indicated antibodies.

### RNA extraction and real-time qRT-PCR

Total cellular RNA was isolated using TRIzol reagent according to the manufacturer's instructions (Invitrogen Corp.). Equal amounts of total RNA (2 µg) were reverse transcribed using TaqMan Reverse Transcription Reagents (Applied Biosystems), and the mRNA levels of the indicated genes were analyzed in triplicate using SYBR Green Master Mix and a Chromo-4 real-time RT-PCR instrument (MJ Research). The mRNA levels were normalized to β-actin (internal control) and gene expression was presented as fold changes. The primer sequences used in the PCR reactions are listed in Table 1.

**Table 1:** Primer sequences.

Genes	GenBank #	Forward (5′-3′)	Reverse (5′-3′)	Size (bp)
*Osterix*	NM_130458	ACCAGGTCCAGGCAACAC	GCAGTCGCAGGTAGAACG	373
*Runx2*	NM_009820	CCACCACTCACTACCACACG	TCAGCGTCAACACCATCATT	250
*Smurf1*	NM_029438	AGTTCGTGGCCAAATAGTGG	GTTCCTTCGTTCTCCAGCAG	99
*Smurf2*	NM_025481	GTGAAGAGCTCGGTCCTTTG	AGAGCCGGGGATCTGTAAAT	114
*β-actin*	NM_007393	CTGGCACCACACCTTCTACA	GGTACGACCAGAGGCATACA	190

### Construction of Osx promoter-luciferase reporter (Osx-Luc)

A 791-nucleotide promoter sequence corresponding to −700 to +91 region of the *Osx* gene was PCR amplified from mouse genomic DNA. The primer sequences used in PCR amplification are: 5′-ATAGCGGTACCTTGTTCCTTTTCCCTCCTGTTC-3′ (forward), and 5′- GTATCAAGCTTCTGGGGACCGGGTCCCAAG -3′ (reverse). A Kpn I and a Hind III restriction site (underlined) were included in the forward and reverse primers, respectively. The resulting PCR product was digested with Kpn I and Hind III and inserted into the corresponding cloning sites in the pGL3 Basic luciferase reporter vector (Promega, Madison, WI).

### Transient transfection and luciferase assays

Osx-Luc was cotransfected with the indicated amounts of expression vectors and an internal control plasmid (pRL-null) into C3H10T1/2 cells using Lipofectamine Plus reagents (Invitrogen Corp.). Total amount of DNA in each transfection was kept constant by adding empty vector DNA (pcDNA3). Twenty-four hours after transfection, the cells were lysed and luciferase activity measured with separate substrates to detect the luciferase (firefly) in the pGL3-basic plasmid and to the second luciferase (Renilla) encoded by the pRL-null vector (dual luciferase assay kit, Promega) as previously described [24]. Luciferase values were normalized to *Renilla* luciferase to correct variations in transfection efficiency.

### Statistics analysis

Data were analyzed with InStat Software (version 3.06, GraphPad Software Inc.) using one-way analysis of variance (ANOVA) followed by Bonferroni multiple comparison test. A *p* value less than 0.05 (*p*<0.05) was considered to be statistically significant. Fold differences are indicated and are presented as mean ± SD.

## Supporting Information

Figure S1 **Continuous glucocorticoid treatment inhibits MSC osteogenic differentiation.** MSC-GFP and MSC-GILZ cells were cultured in OS without or with 100 nM dexamethasone (Dex) for the first 2, 4, 8, or 12 days and then switched to OS without Dex for the remaining days indicated. Cells were stained with ARS on day 22.
(TIF)
