# Supplementary figures and images for: Glucocorticoid-Induced Leucine Zipper (GILZ) Antagonizes TNF-α Inhibition of Mesenchymal Stem Cell Osteogenic Differentiation
Source: PLoS One. 2012 Mar 2;7(3):e31717. doi: 10.1371/journal.pone.0031717 (PMC3292550; doi:10.1371/journal.pone.0031717)

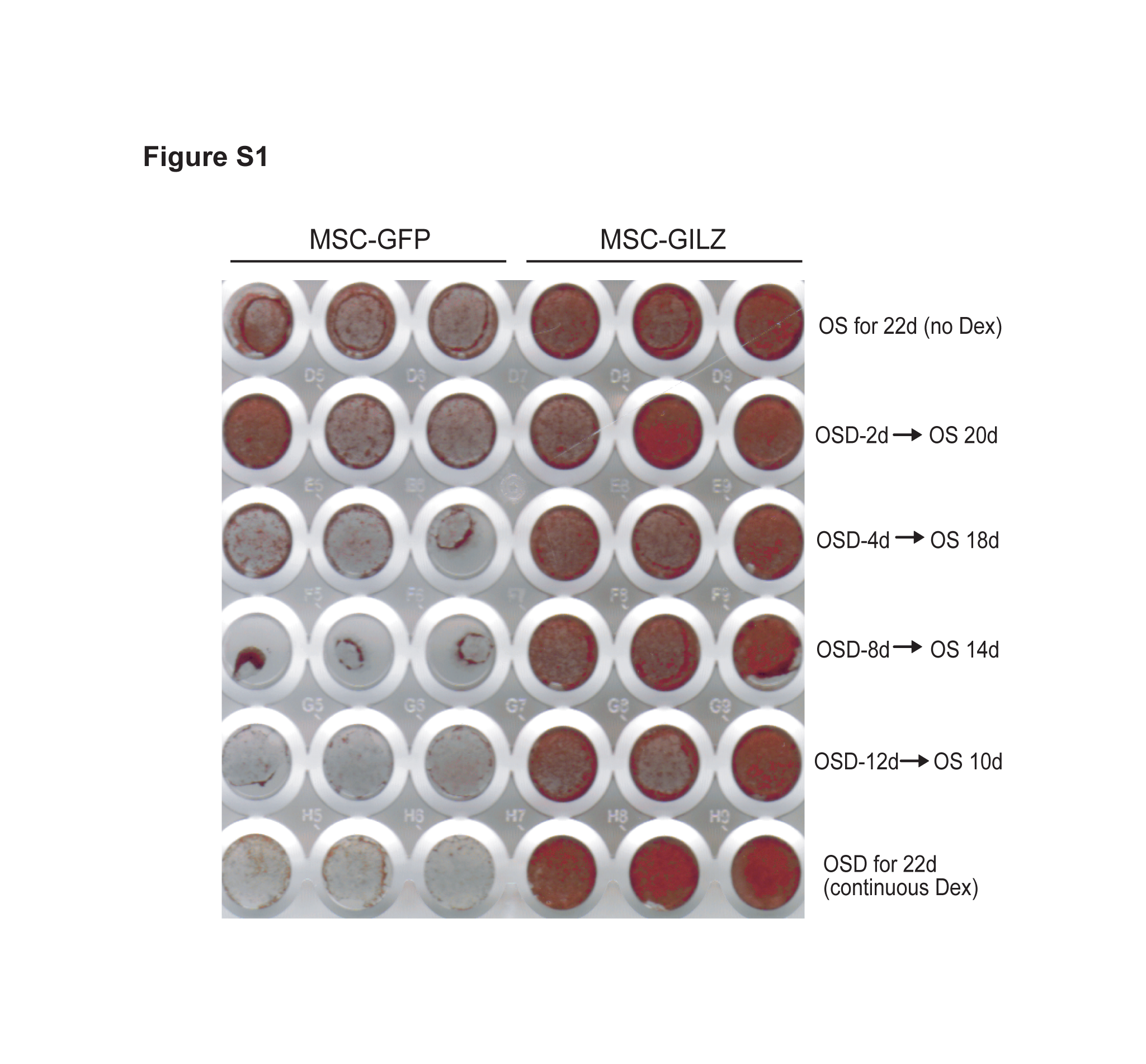

Supplement: Figure S1 — Continuous glucocorticoid treatment inhibits MSC osteogenic differentiation. MSC-GFP and MSC-GILZ cells were cultured in OS without or with 100 nM dexamethasone (Dex) for the first 2, 4, 8, or 12 days and then switched to OS without Dex for the remaining days indicated. Cells were stained with ARS on day 22. (TIF) [file pone.0031717.s001.tif]
